# A New Semi-Quantitative Parameter to Assess Functionally Significant Coronary Disease Using Myocardial Contrast Echocardiography

**DOI:** 10.31083/j.rcm2512431

**Published:** 2024-12-05

**Authors:** Jili Long, Jingru Lin, Jia Tao, Hao Wang

**Affiliations:** ^1^Department of Echocardiography, State Key Laboratory of Cardiovascular Disease, Fuwai Hospital, National Center for Cardiovascular Diseases, Chinese Academy of Medical Sciences and Peking Union Medical College, 100006 Beijing, China

**Keywords:** myocardial contrast echocardiography, quantitative flow ratio, coronary disease

## Abstract

**Background:**

Quantitative flow ratio (QFR) can identify functionally significant coronary disease non-invasively. Myocardial contrast echocardiography (MCE) is a non-invasive and effective procedure for detecting abnormalities in hemodynamic coronary artery stenosis. Currently, there is no research confirming the correlation between MCE and QFR. This study aims to compare the capacity of the perfusion index (PI) from MCE to diagnose functionally significant coronary disease in patients with chest pain. The investigators use QFR as the gold standard for comparison.

**Methods:**

112 patients referred for coronary angiography (CAG) due to suspicion of coronary artery disease (CAD) were included. 64 patients with functionally significant coronary disease were diagnosed. 48 patients were defined as CAD without functionally significant coronary disease. MCE was performed 24 h before angiography. PI was calculated for each triggering interval by adding the perfusion scores of segments and dividing by the number of segments. Logistic regression analyses were performed to evaluate the association among functionally significant coronary disease, echocardiographic and clinical parameters. Spearman correlation analysis was used to investigate the correlation between PI and QFR. A receiver operating characteristic (ROC) curve was used to assess the capability of echocardiographic and clinical parameters to diagnose functionally significant coronary disease.

**Results:**

Patients with functionally significant coronary disease had the worse perfusion in MCE compared with those without functionally significant coronary disease. In multivariable logistic regression analysis, global perfusion index (GPI) (OR: 43.409, *p* < 0.001) was associated with functionally significant coronary disease in patients with CAD. Based on the Spearman correlation analysis. Left anterior descending artery (LAD)-PI showed a strong negative correlation with LAD-QFR (r = –0.652, *p* < 0.01). ROC curves showed LAD-PI to be superior to GPI, left circumflex artery PI (LCX-PI) and right coronary artery PI (RCA-PI) in identifying functionally significant coronary disease.

**Conclusions:**

The PI derived from MCE has diagnostic value for functionally significant coronary disease with QFR ≤0.80 in 1 or more vessels, with LAD-PI showing the highest diagnostic efficiency. GPI is independently associated with functionally significant coronary disease, but among the branch PIs, LAD-PI has the highest diagnostic efficiency.

## 1. Introduction

Cardiovascular disease is one of the leading causes of mortality world-wide [1]. Coronary artery disease (CAD) is the leading cause of mortality globally, accounting for 8.9 million deaths each year [2]. Previous clinical studies have shown that the incidence of adverse events (death, non-fatal myocardial infarction) in patients with CAD is closely related to the degree of functional coronary stenoses [3, 4, 5]. Due to a lack of precise assessment tools, some patients with CAD are either not optimally treated or overtreated, wasting medical resources and causing significant economic impact. Accurately evaluating the function of coronary artery stenosis is crucial and a current focus of research in this field.

Quantitative flow ratio (QFR) is a novel diagnostic method for detecting functional coronary artery stenosis and induction of hyperemia without the use of pressure wires [6]. It eliminates the need for vasodilators and guidewires, utilizing coronary angiography (CAG) imaging for three-dimensional reconstruction and hemodynamic analysis. This method rapidly provides flow reserve scores, which show a high correlation with fractional flow reserve (FFR) [6, 7, 8]. Presently, QFR has received certification from the U.S. Food and Drug Administration (FDA), and the European Conformité Européenne, establishing it as the “new standard” for the evaluation of functionally significant coronary disease defined by QFR ≤0.80.

Although the QFR holds significant promise in the clinical application for assessing functionally significant CAD, it remains an invasive procedure and has not yet been widely adopted in primary healthcare facilities. Despite the existence of several non-invasive methods for diagnosing myocardial ischemia in current clinical practice, a considerable number of patients undergoing CAG nationwide still do not meet the criteria for revascularization. Therefore, the pursuit of an accurate, non-invasive, easy-to-operate, and cost-effective method for assessing functionally significant coronary disease continues to be a topic of interest for researchers.

Myocardial contrast echocardiography (MCE) can observe the perfusion of the left ventricular (LV) myocardium in real-time and has the advantages of being convenient and low-cost [9]. It has taken on an increasingly important role in the treatment and diagnosis of heart disease. MCE at rest is a non-invasive and effective method for detecting abnormalities in the coronary microcirculation, which helps in the clinical diagnosis, risk assessment, and treatment of early and asymptomatic CAD [10]. A study has shown that MCE and single-photon emission computed tomography (SPECT) have similar effectiveness in detecting CAD, with a sensitivity and specificity of 81% and 83% [11]. Additionally, MCE assesses ventricular wall motion and cardiac function along with myocardial perfusion [12].

Currently, there is no research confirming the correlation between MCE and QFR. This study aims to compare the capacity of the perfusion index (PI) from MCE to diagnose functionally significant coronary disease in patients with chest pain. The investigators use QFR as the gold standard for comparison. It attempts to offer a non-invasive, clinically effective imaging method for pre-angiographic screening of coronary artery stenoses.

## 2. Materials and Methods

### 2.1 Study Population

This research was conducted in a single, tertiary coronary care center with 112 patients who had undiagnosed unknown ischemic heart disease but were referred with angina pectoris and were scheduled to undergo MCE, CAG and QFR assessment. Patients were excluded with the following criteria: age <18 years; abnormal baseline wall motion; previous myocardial infarction; a history of coronary artery bypass surgery, unstable angina, second-or third-degree atrioventricular block, chronic obstructive pulmonary disease, severe valvular heart disease, severe ventricular arrhythmias, severe liver and kidney dysfunction; history of allergy to echocardiography contrast agents, any intracardiac shunt procedures, pregnancy, and breastfeeding. All patients underwent two-dimensional (2D) echocardiography and MCE followed by CAG.

All study participants or their legal representatives provided written informed consent. The study complied with the Declaration of Helsinki and was approved by the Ethics Committees of Fuwai Hospital (no. 2021-1429).

### 2.2 Conventional Echocardiography

All patients underwent a comprehensive conventional 2D echocardiographic assessment at rest in accordance with the latest guidelines [13], utilizing a commercially accessible ultrasound platform (EPIQ 7C, Philips Healthcare, Andover, MA, USA). Echocardiographic images were analyzed offline using the Qlab 13.0 (Philips Healthcare, Andover, MA, USA). Left ventricular ejection fraction (LVEF) was measured using Simpson’s biplane method. LV mass was assessed using a 2D approach, and LV mass index (LVMI) was calculated by dividing LV mass by body surface area (BSA). Pulsed-wave Doppler in the apical four-chamber view assessed mitral inflow, measuring peak E and A wave velocities.

### 2.3 Myocardial Contrast Echocardiography (MCE)

MCE was performed in the three standard apical views (four-, two-, and three-chamber). The contrast agent used was the commercially accessible perfluoropropane-albumin Microspheres contrast (Yangtze River Pharmaceutical Group, Taizhou, China). Contrast agents were applied during real-time imaging as a continuous intravenous infusion (EPIQ 7C, Philips Healthcare, Andover, MA, USA) at an extremely low mechanical index (<0.2). Adjustments to time gain compensation and 2D gain settings were made as advised to eliminate tissue signals prior to contrast administration, and these settings were maintained consistently during the study. Continuous infusion was paired with a “Flash” trigger at left ventricular end-systole (frame rate 5–15 fps, mechanical index (MI) 0.8–1.2), collecting images from at least one cardiac cycle before “Flash” and 15 cycles after “Flash” in each apical window [14].

Microvascular perfusion (MVP) analysis was done by two blinded investigators during the myocardial replenishment period using the 18-segment American Heart Association model. For MVP assessment, segmental perfusion scoring was performed (1 = normal MVP [nMVP], 2 = delayed MVP [dMVP], 3 = microvascular obstruction [MVO]). A PI was calculated for each triggering interval by adding the perfusion scores of segments and dividing by the number of segments [15]. The segments were divided into the left anterior descending artery (LAD), left circumflex artery (LCX) and right coronary artery (RCA) vascular territories. Global perfusion index (GPI) = total perfusion scoring/18. MVP was classified as nMVP when full myocardial segment contrast replenishment occurred within 4 seconds post high-mechanical index impulse. A perfusion defect persisting beyond 4 seconds yet achieving full replenishment by 10 seconds was defined as dMVP. MVO was characterized by the presence of a lasting defect after achieving plateau intensity [16].

### 2.4 Online QFR Assessment

The QFR system (AngioPlus, Pulse Medical Imaging Technology, Shanghai Co., Ltd., Shanghai, China) received angiographic images taken at two distinct angles (25°) through a local network [17]. The QFR value was calculated after three-dimensional reconstruction of coronary arteries using the QFR system. Critical restenosis lesions were assessed functionally by two trained medical technicians using the QFR measurement software blindly, with their assessments averaged for accuracy. Functionally significant coronary disease was defined by QFR ≤0.80 in 1 or more vessels. For each patient, QFR values were calculated separately for the LAD, LCX, and RCA. The lowest QFR value among the three coronary arteries (LAD, LCX, and RCA) was selected as the patient’s final QFR value.

### 2.5 Statistical Analysis

According to the normal distribution, continuous variables were reported as mean ± SD or median with interquartile ranges. Categorical variables were reported as frequencies and percentages. The Shapiro-Wilk test was used to assess the normal distribution. We compared continuous data with either the Mann-Whitney U test or the Student’s *t*-test, and analyzed categorical data using the chi-square or Fisher’s exact tests. Pearson or Spearman correlation was applied to continuous variables, and univariate logistic regression identified factors associated with functionally significant coronary disease. After removing variables with collinearity (either Pearson’s or Spearman’s correlation ≤0.60 or variance inflation factor >10), all relevant echocardiographic and clinical variables were included in the multivariate analysis. Receiver operating characteristic (ROC) curves were generated using QFR as the gold standard to compare the area under the curve (AUC), sensitivity, specificity, 95% confidence intervals, and cutoff values for various parameters. Statistical analyses were conducted with SPSS version 25.0 (IBM, Armonk, NY, USA), MedCalc, version. All tests were two-sided, with *p* values less than 0.05 indicating statistical significance.

## 3. Results

### 3.1 Characteristics of the Study Population

The study included 112 participants, with 88 males and 24 females. All participants underwent CAG and QFR assessments, with details provided in Table 1. There were 48 patients (42.9%) with a QFR >0.8, while 64 patients (57.1%) had functionally significant coronary disease with QFR ≤0.8. No statistical differences were detected between patients with QFR >0.8 and those with QFR ≤0.8 in terms of gender, age, BSA, blood pressure, and coronary dominance. However, the use of statin medication was considerably higher in the group with functionally significant coronary disease compared to the control group (QFR >0.8), indicating a statistically significant difference.

**Table 1.** **Characteristics of study population**.

Characteristic		Patients with QFR >0.8 (n = 48, 42.9%)	Patients with QFR ≤0.8 (n = 64, 57.1%)	*p* value
Age, years		57 ± 8.6	58 ± 9.7	0.59
Gender, male		35 (72.9%)	53 (82.8%)	0.21
BSA, m^2^		1.83 ± 0.19	1.84 ± 0.155	0.84
BMI (kg/m^2^)		25.36 ± 2.56	25.66 ± 3.0	0.58
Medical history				
	Hypertension	28 (58.3%)	36 (56.3%)	0.83
	Diabetes mellitus	15 (31.3%)	23 (35.9%)	0.60
	Hyperlipidemia	40 (83.3%)	51 (79.7%)	0.63
	Smoking history	25 (52.1%)	32 (50.0%)	0.83
	Drinking history	16 (33.3%)	23 (35.9%)	0.78
Medications				
	β-blocker	15 (36.6%)	26 (40.6%)	0.31
	ACE inhibitors/ARBs	10 (20.8%)	11 (17.2%)	0.63
	CCBs	5 (10.4%)	11 (17.2%)	0.31
	Statins	33 (68.8%)	54 (84.4%)	0.05
	Antiplatelet drugs	38 (79.2%)	53 (82.8%)	0.63
	Nitrates	18 (37.5%)	26 (40.6%)	0.74
Coronary dominance				
	Right	44 (91.7%)	61 (95.3%)	>0.999
	Left	2 (4.2%)	3 (4.7%)	>0.999
	Balanced	2 (4.2%)	0 (0%)	>0.999
Vessel involved				
	0 vessel	9 (18.8%)	1 (1.1%)	<0.001
	Single vessel	14 (29.2%)	9 (9.7%)	<0.001
	Two vessels	14 (29.2%)	19 (20.4%)	<0.001
	Three vessels	11 (22.9%)	35 (68.8%)	<0.001
Culprit vessel				
	LMCA	6 (12.5%)	20 (31.3%)	0.02
	LAD	24 (50%)	44 (68.8%)	0.44
	RCA	13 (27.1%)	33 (51.6%)	0.01
	LCX	13 (27.1%)	40 (62.5%)	<0.001

BMI, body mass index; BSA, body surface area; CCBs, calcium channel blockers; LAD, left anterior descending artery; LCX, left circumflex artery; LMCA, left main coronary artery; RCA, right coronary artery; QFR, quantitative flow ratio; ACE, angiotensin-converting enzyme; ARBs, angiotensin receptor blockers.

### 3.2 Biochemical Test Results 

Biochemical test results are presented in Table 2. Compared with patients without functionally significant coronary disease, patients with functionally significant coronary disease had a significantly higher high-sensitivity cardiac troponin I (hs-cTnI) (*p* = 0.011), with an average value of 0.85 ± 0.331 ng/mL There were no statistically significant differences in blood glucose, blood lipids, B-type natriuretic peptide (BNP), and thyroid function between the two groups.

**Table 2.** **Biochemical test results**.

Parameters	Patients with QFR >0.8 (n = 48, 42.9%)	Patients with QFR ≤0.8 (n = 64, 57.1%)	*p* value
HbA1C	6.24 ± 0.96	6.50 ± 1.424	0.32
GLU	7.18 ± 4.50	7.34 ± 2.99	0.82
TG	1.73 ± 1.215	1.70 ± 0.881	0.89
CHOL	4.40 ± 1.363	4.15 ± 0.937	0.29
HDLC	1.33 ± 0.396	1.23 ± 0.296	0.12
LDLC	2.54 ± 1.071	2.37 ± 0.849	0.37
hs-cTnI	0.29 ± 1.133	0.85 ± 3.301	0.01
MYO	42.7 ± 22.167	55.89 ± 87.35	0.31
NTPRO-BNP	109.43 ± 126.492	180.59 ± 290.478	0.12
FT3	3.36 ± 0.35	3.30 ± 0.35	0.36
FT4	1.30 ± 0.154	1.25 ± 0.15	0.12
T3	1.18 ± 0.20	1.17 ± 0.18	0.71
T4	8.79 ± 1.67	8.72 ± 1.92	0.83

HbA1C, hemoglobin A1C; GLU, glucose; TG, triglycerides; CHOL, cholesterol; HDLC, high-density lipoprotein cholesterol; LDLC, low-density lipoprotein cholesterol; MYO, myoglobin; QFR, quantitative flow ratio; hs-cTnI, high-sensitivity cardiac troponin I; NTPRO-BNP, N-terminal pro b-type natriuretic peptide; FT3, free triiodothyronine; FT4, free thyroxine; T3, triiodothyronine; T4, thyroxine.

### 3.3 Conventional Echocardiographic and Myocardial Perfusion Parameters

Conventional echocardiographic parameters are presented in Table 3. Patients with functionally significant coronary disease (QFR ≤0.8) had greater interventricular septum thickness (IVS), left ventricular posterior wall thickness (LVPWd), and LVMI compared to patients without functionally significant coronary disease, with *p*-values of 0.007, 0.038, and 0.006, respectively. There were no statistically significant differences in other routine echocardiographic parameters between patients with and without functionally significant coronary disease.

**Table 3.** **Conventional echocardiography, perfusion index**.

Parameters	Patients with QFR >0.8 (n = 48, 42.9%)	Patients with QFR ≤0.8 (n = 64, 57.1%)	*p* value
LVIDd, mm	48 ± 3.70	64 ± 3.59	0.20
LVIDs, mm	29 ± 3.14	30 ± 3.24	0.65
IVS, mm	9.58 ± 1.555	10.50 ± 1.860	0.01
LVPWd, mm	8.9 ± 0.973	9.31 ± 1.082	0.04
LVMI, g/m^2^	80 ± 17.438	90 ± 19.286	0.01
E velocity, m/sec	75 ± 16.933	72 ± 15.389	0.33
A velocity, m/sec	48 ± 15.501	83 ± 16.789	0.88
E/A ratio	0.91 ± 0.210	0.88 ± 0.175	0.42
Rest LVEF, %	65 ± 3.971	66 ± 4.823	0.65
GPI	1.04 ± 0.20	1.72 ± 0.52	<0.001
LAD-PI	1.125 ± 0.29	1.920 ± 0.56	<0.001
LCX-PI	1.09 ± 0.203	1.59 ± 0.63	<0.001
RCA-PI	1.04 ± 0.10	1.29 ± 0.48	<0.001

LVIDd, LV internal dimension diastole; LVIDs, LV internal dimension systole; IVS, interventricular septum; LVPWd, left ventricle posterior wall diastole; LVMI, LV mass index; GPI, global perfusion index; LAD, left anterior descending artery; LCX, left circumflex artery; RCA, right coronary artery; QFR, quantitative flow ratio; PI, perfusion index; LV, left ventricular; LVEF, left ventricular ejection fraction.

The semi-quantitative assessment results of MCE are shown in Table 3. Compared with patients without functionally significant coronary disease, patients with functionally significant coronary disease had a significantly higher GPI, with an average value of 1.72 ± 0.52 (*p* < 0.001). In addition, the PI of the other three major branches (LAD-PI, LCX-PI, RCA-PI) were also significantly higher than those in the group without functionally significant coronary disease (*p* < 0.001, for all).

### 3.4 Correlations among Statins, hs-cTnI, and Echocardiographic Parameters

LVPWd and LVMI all showed strong correlations with IVS (r = 0.624, 0.771, respectively, *p* < 0.01 for all; Table 4) but weak correlations with hs-cTnI (r = –0.01, 0.004, respectively, *p* > 0.05 for all; Table 4). GPI also showed good correlations with LAD-PI (r = 0.769, *p* < 0.01) and LCX-PI (r = 0.600, *p* < 0.01; Table 4). hs-cTnI also showed weak correlations with GPI and Stains (r = 0.04, –0.11, respectively, *p* > 0.05 for all; Table 4).

**Table 4.** **Correlations among statins, hs-cTnI, and echocardiographic parameters**.

Parameters	Statins	hs-cTnI, ng/mL	IVS, mm	LVPWd, mm	LVMI, g/m^2^	GPI	LAD-PI	LCX-PI	RCA-PI
Statins	1.00	–0.11	0.07	0.09	0.10	0.15	0.11	0.09	0.11
hs-cTnl, ng/mL	–0.11	1.00	0.04	–0.01	0.04	0.04	0.05	0.05	–0.07
IVS, mm	0.07	0.04	1.00	0.624**	0.771**	0.292**	0.359**	0.06	0.212*
LVPWd, mm	0.09	–0.01	0.624**	1.00	0.644**	0.283**	0.290**	0.14	0.215*
LVMI, g/m^2^	0.10	0.04	0.771**	0.644**	1.00	0.343**	0.380**	0.13	0.240*
GPI	0.15	0.04	0.292**	0.283**	0.343**	1.00	0.769**	0.600**	0.418**
LAD-PI	0.11	0.05	0.359**	0.290**	0.380**	0.769**	1.00	0.437**	0.403**
LCX-PI	0.09	0.05	0.06	0.14	0.13	0.600**	0.437**	1.00	0.495**
RCA-PI	0.11	–0.07	0.212*	0.215*	0.240*	0.418**	0.403**	0.495**	1.000**

** *p* < 0.01, * *p* < 0.05; hs-cTnI, high-sensitivity cardiac troponin I; IVS, interventricular septum; LVPWd, left ventricular posterior wall diameter; LVMI, left ventricular mass index; GPI, global perfusion index; LAD-PI, left anterior descending artery perfusion index; LCX-PI, left circumflex artery perfusion index; RCA-PI, right coronary artery perfusion index.

### 3.5 Binary Logistic Regression Analyses for the Detection of Functional Significant Coronary Disease 

In the univariate logistic regression analysis, the following variables were correlated with functionally significant coronary disease: Stains, hs-cTnI, IVS, LVMI, GPI (Table 5). In the multivariate logistic regression analysis, GPI (OR = 43.409, *p* < 0.001) was correlated with functionally significant coronary disease after adjusting for the potential confounders and excluding parameters (LVPWd, LAD-PI, LCX-PI, RCA-PI) with collinearity (Table 5).

**Table 5.** **Univariate and multivariate logistic regression analysis showing parameters association with coronary functinal steneosis CAD**.

Parameters	Univariate analysis		Multivariable analysis	
	OR (95% CI)	*p* value	OR (95% CI)	*p* value
Statins	2.455 (0.988–6.097)	0.053		
hs-cTnI, ng/mL	1.122 (0.896–1.404)	0.317		
IVS, mm	1.383 (1.082–1.767)	0.01		
LVMI, g/m^2^	1.031 (1.008–1.055)	0.008		
GPI	48.406 (10.686–219.270)	<0.001	43.409 (9.260–203.501)	<0.001

hs-cTnI, high-sensitivity cardiac troponin I; IVS, interventricular septum; LVMI, left ventricular mass index; GPI, global perfusion index; CAD, coronary artery disease; OR, odds ratio; CI, confidence interval.

### 3.6 Correlations among LAD-PI, LCX-PI, RCA-PI and LAD-QFR, LCX-QFR, RCA-QFR

**Fig. 1.**
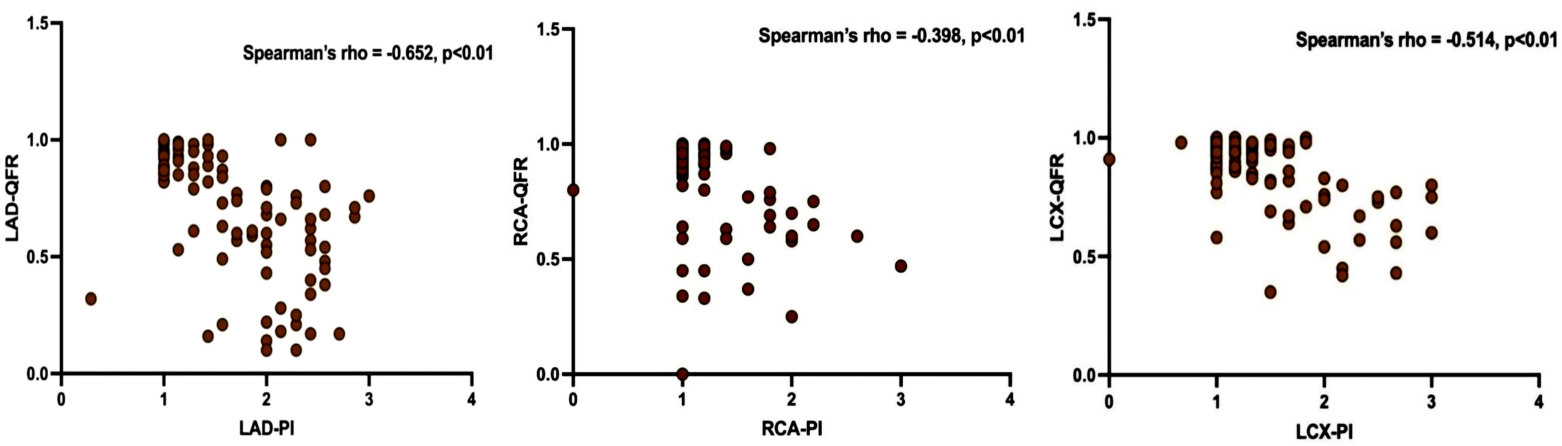
**Scatter plot**. PI versus QFR. PI, perfusion index; QFR, quantitative flow ratio; LAD-PI, left anterior descending artery perfusion index; LCX-PI, left circumflex artery perfusion index; RCA-PI, right coronary artery perfusion index; LAD-QFR, left anterior descending artery quantitative flow ratio; LCX-QFR, left circumflex artery quantitative flow ratio; RCA-QFR, right coronary artery quantitative flow ratio.

Based on the Spearman correlation analysis, there is a significant negative correlation between LAD-PI and LAD-QFR, LCX-PI and LCX-QFR, RCA-PI and RCA-QFR (r = –652, –0.514, –0.398, respectively, *p* < 0.01 for all; Fig. 1). LAD-PI showed good correlations with LAD-QFR (r = –0.652, *p* < 0.01). RCA-PI showed weak correlations with RCA-QFR (r = –0.398, *p* < 0.01).

### 3.7 Receiver Operating Characteristic Curve Analysis for the Detection of CAD with Coronary Functional Steneosis 

**Fig. 2.**
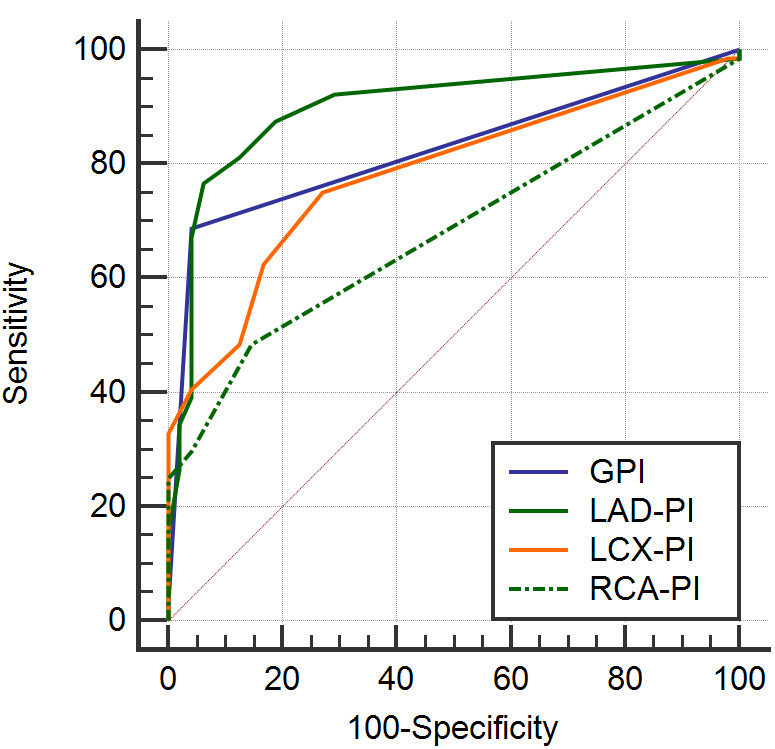
**Receiver operating characteristics curve analysis**. Receiver operating characteristics curve analysis to predict functionally significant coronary disease. LAD-PI, left anterior descending artery perfusion index; LCX-PI, left circumflex artery perfusion index; RCA-PI, right coronary artery perfusion index; GPI, global perfusion index.

According to the ROC analysis, LAD-PI had the largest AUC (AUC = 0.898) to detect functionally significant coronary disease in CAD among LAD-PI, LCX-PI and RCA-PI (Fig. 2). The cutoff of GPI, LAD-PI, LCX-PI, and RCA-PI was 1.50, 1.50, 1.083 and 1.100, respectively, and the sensitivity and specificity of MCE for detecting functionally significant coronary disease were 68.70% and 95.80%, 76.56% and 93.75%, 75.00% and 72.92%, 48.44% and 85.42%, respectively (Table 6).

**Table 6.** **Performance of PI for detecting functionally significant coronary disease**.

Parameters	AUC	*p* value	95% CI		Cutoff-value	Sensitivity	Specificity
			Upper limit	Lower limit			
GPI	0.824	<0.01	0.740	0.889	1.500	68.70	95.80
LAD-PI	0.898	<0.01	0.826	0.947	1.500	76.56	93.75
LCX-PI	0.782	<0.01	0.694	0.854	1.083	75.00	72.92
RCA-PI	0.679	0.001	0.585	0.764	1.100	48.44	85.42

GPI, global perfusion index; LAD-PI, left anterior descending artery perfusion index; LCX-PI, left circumflex artery perfusion index; RCA-PI, right coronary artery perfusion index; AUC, area under the curve; CI, confidence interval.

## 4. Discussion

In this study, we illustrated the diagnostic potential of PI for detection of functionally significant coronary disease in subjects with angina pectoris. LAD-PI was the optimal parameter to identify functionally significant coronary disease among GPI, LCX-PI and RCA-PI, subsequent to GPI.

In our study, compared to the control group, patients with coronary heart disease who had functionally significant coronary disease show a significant increase in IVS and LVMI. The results are consistent with previous studies [18, 19]. When the coronary arteries undergo functional stenosis, the myocardium increases its mass to maintain adequate blood flow through the area of stenosis to satisfy the body’s needs. This adaptive thickening is most commonly observed in the LV, especially in the septum [20, 21]. While changes in IVS can indicate alterations in heart muscle thickness and potentially suggest pathological conditions leading to ischemia, they do not directly measure blood flow or perfusion [22]. MCE employs contrast agents to enhance the echocardiographic image, allowing for a detailed visualization of myocardial perfusion [23].

**Fig. 3.**
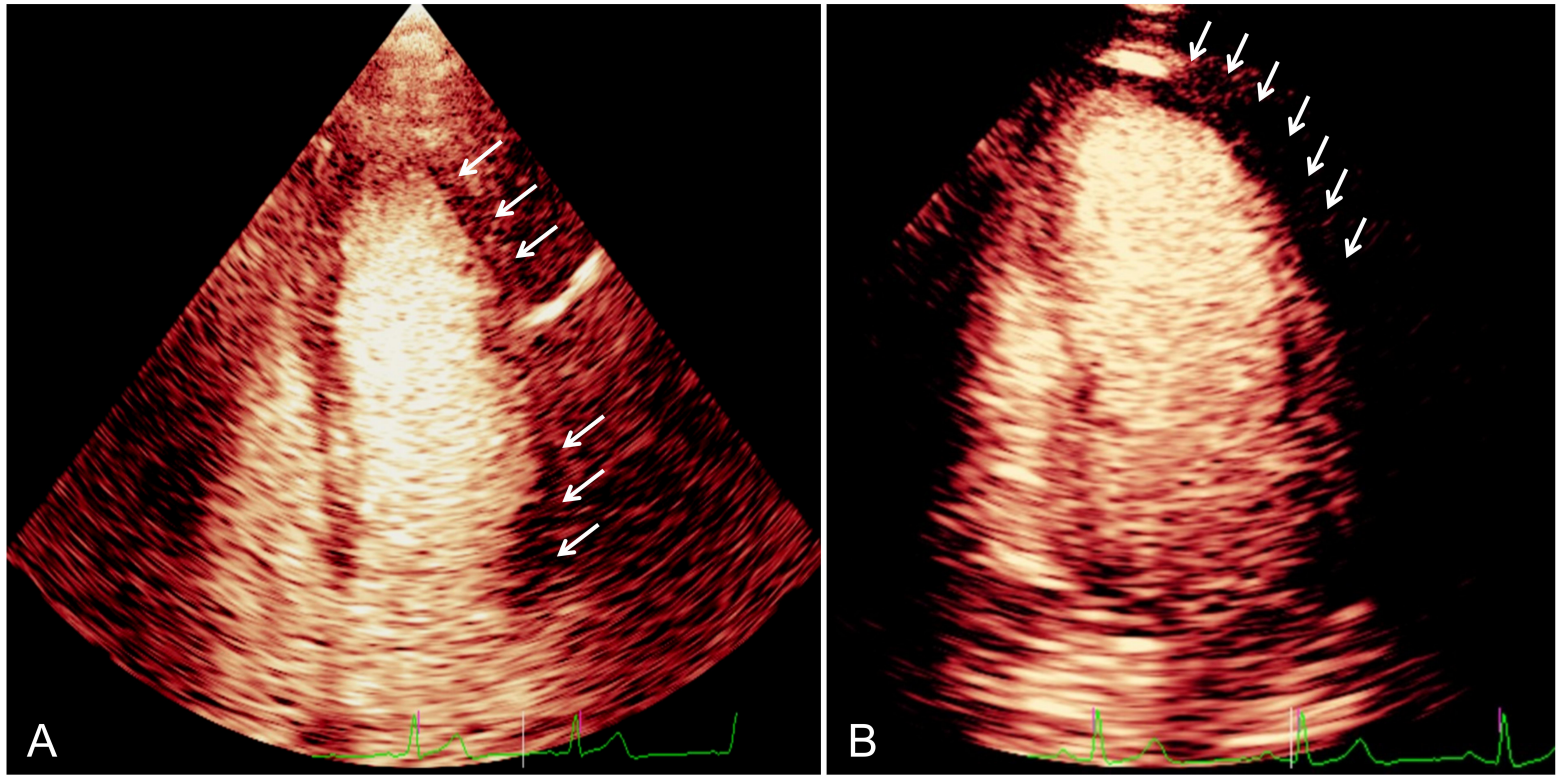
**The MCE findings in patients with QFR <0.8**. (A) Example of a 53-year-old man with 90% stenosis in LCX and QFR 0.77, apical four-chamber view on MCE with dMVP (arrows) at the apex and lateral wall. (B) Example of a 49-year-old man with 80% stenosis in LAD and QFR 0.21, apical two-chamber view on MCE with MVO (arrows) at the apex and anterior wall. MCE, myocardial contrast echocardiography; QFR, quantitative flow ratio; LCX, left circumflex artery; LAD, left anterior descending artery; dMVP, delayed microvascular perfusion; MVO, microvascular obstruction.

MCE is widely used to detect CAD in clinical practice. However, the current clinical examination using MCE is subjective, heavily operator-dependent, and time-consuming [24, 25]. Thus, a new semiquantitative scoring system validated by our group was used to evaluate contrast intensity at 15 cardiac cycles after Flash in the 18-segment LV model. This new semiquantitative parameter was developed based on previously published methods with good reproducibility [15, 26]. However, those previously published methods provided only a few points on the reperfusion curve, and the insufficiently detailed scoring system could lead to a loss of diagnostic information. In our study, patients with functionally significant coronary disease had the worse perfusion in MCE compared with those without functionally significant coronary disease with a QFR <0.8. Two examples showing MCE perfusion abnormalities in patients with QFR <0.8 are presented in Fig. 3. Our study reveals significant correlations between the PI and QFR. Increased perfusion abnormalities, as indicated by higher PI values, is associated with lower QFR values. LAD-PI has shown a stronger correlation with LAD-QFR compared to other branch PIs. This could be partially influenced by the patient population, where most patients had LAD-dominant disease.

The effectiveness of MCE in detecting abnormalities in coronary microcirculation has been documented in the literature, highlighting its value in assessing myocardial perfusion [27, 28]. However, the diagnostic capability of MCE in identifying functionally significant coronary disease has not been explored before our study. We observed that MCE has a good predictive value for abnormalities in QFR. LAD-PI has the highest diagnostic value for detecting coronary functional disease with QFR <0.8. This is because the LAD artery supplies a significant portion of the myocardium, including the anterior wall and apex, which are critical for cardiac function. Thus, impairments in LAD perfusion are more detectable and diagnostically significant [29, 30]. Previous study has shown that MCE can predict abnormalities in coronary flow reserve (CFR) [31]. Similar to QFR, CFR implies that a coronary stenosis is hemodynamically significant [32]. These studies demonstrate that MCE is capable of not only identifying macroscopic coronary artery lesions, but also assessing the hemodynamic significance of a coronary stenosis.

For assessment of post-percutaneous coronary intervention (PCI) perfusion at the microvascular level, abnormal microvascular flow observed by MCE is associated with a high rate of adverse outcomes. Reduced or absent MVP in acute ST-segment–elevation myocardial infarction is common and associated with significantly worse outcomes for microembolization [16, 33, 34]. A new semi-quantitative parameter, PI was calculated from MVP in our study. PI also can play an important role in assessing abnormal microvascular flow.

In summary, the findings of this study support the value of using non-invasive imaging techniques in the diagnosis and management of CAD through MCE. By introducing a new semi-quantitative parameter for assessing MCE images, we have not only improved the accuracy of diagnosing functionally significant coronary disease but also provided new insights for future research in this field. With the ongoing advancements in non-invasive diagnostic technologies, we look forward to their increased role in the early diagnosis and personalized treatment of cardiovascular diseases.

## 5. Study Limitations

This study lacks original data on the reproducibility of MCE, and differences in operation and equipment may affect result consistency, contributing to the observed scatter. Future research should consider including reproducibility assessments or adopting standardized procedures to enhance reliability. Being a single-center study, the relatively small sample size may limit the generalizability of our findings. Therefore, our conclusions need to be validated in larger, multi-center studies. Similar to SPECT used to evaluate microvascular function, MCE was not confirmed against a gold standard for microvascular dysfunction in patients. However, these two techniques are closely correlated when quantifying hyperemic responses [24]. Furthermore, future research should consider including MCE evaluations with exercise or pharmacological stress testing, which could provide more comprehensive information about the myocardial perfusion status. It’s noteworthy that quantitative analysis of contrast opacification might have improved the separation of groups even further [15].

## 6. Conclusions

Our study reveals significant correlations between the PI and QFR. MCE provides incremental diagnostic value for detecting functionally significant coronary disease in patients with angina pectoris. Furthermore, LAD-PI demonstrated increased diagnostic potential in identifying functionally significant coronary disease but also provided added value to estimate the probability of functionally significant coronary disease. As a new semi-quantitative parameter in MCE, PI may complement existing diagnostic algorithms and examinations as a reliable noninvasive screening tool before CAG.

## Data Availability

Data generated or analyzed during this study are included in this published article.

## Abbreviations

QFR, quantitative flow ratio; MCE, myocardial contrast echocardiography; CAD, coronary artery disease; CAG, coronary angiography; MVP, microvascular perfusion; MVO, microvascular obstruction; PI, perfusion index; GPI, global perfusion index; LAD, left anterior descending artery; LCX, left circumflex artery; RCA, right coronary artery; LV, Left ventricular; LVIDd, LV internal dimension diastole; LVIDs, LV internal dimension systole; LVMI, LV mass index; LVPWd, left ventricle posterior wall diastole; LVEF, left ventricular ejection fraction; 2D, two-dimensional; IVS, interventricular septum; LAD-QFR, left anterior descending artery quantitative flow ratio; LCX-QFR, left circumflex artery quantitative flow ratio; RCA-QFR, right coronary artery quantitative flow ratio; ROC, receiver operating characteristic; AUC, area under the curve; CI, confidence interval; SPECT, single-photon emission computed tomography; BMI, body mass index; BSA, body surface area; CCBs, calcium channel blockers; GLU, glucose; TG, triglycerides; CHOL, cholesterol; HDLC, high-density lipoprotein cholesterol; LDLC, low-density lipoprotein cholesterol; MYO, myoglobin; HbA1C, hemoglobin A1C; ACE, angiotensin-converting enzyme; ARBs, angiotensin receptor blockers; NTPRO-BNP, N-terminal pro b-type natriuretic peptide; FT3, free triiodothyronine; FT4, free thyroxine; T3, triiodothyronine; T4, thyroxine.
